# Correction: Increase of cell surface vimentin is associated with vimentin network disruption and subsequent stress-induced premature senescence in human chondrocytes

**DOI:** 10.7554/eLife.108668

**Published:** 2025-07-31

**Authors:** Jana Riegger, Rolf E Brenner

**Keywords:** Human

Riegger J, Brenner RE. 2023. Increase of cell surface vimentin is associated with vimentin network disruption and subsequent stress-induced premature senescence in human chondrocytes. *eLife* **12**:e91453. doi: 10.7554/eLife.91453.

Published 19 October 2023

We recently became aware that an image was inadvertently duplicated across two of our publications and immediately informed the editorial office about it. In the following, we would like to explain the circumstances that led to this duplication.

As part of our project, we performed flow cytometric analysis to determine cell surface vimentin (CSV) on human chondrocytes from several donors and later compared the data to the outcome of a chondrogenic differentiation in pellet culture, which was assessed by Safranin-O staining. The image in question shows a Safranin-O staining that indeed represents the outcome of chondrogenesis of the same donor, which was also used for flow cytometric analysis. Since the donor-matched data were not consolidated until several months later, it did not become apparent to us that this particular donor had also been used for a chondrogenic differentiation in a different project. Unfortunately, the same assay type, same donor ID, and same passage led to the inadvertently reuse of the image.

We have since replaced the data in Figure 6g with the results from a different donor. This correction does not affect the conclusions of our study.

The corrected Figure 6 with updated panel g (leftmost image) is shown here:

**Figure 1:**
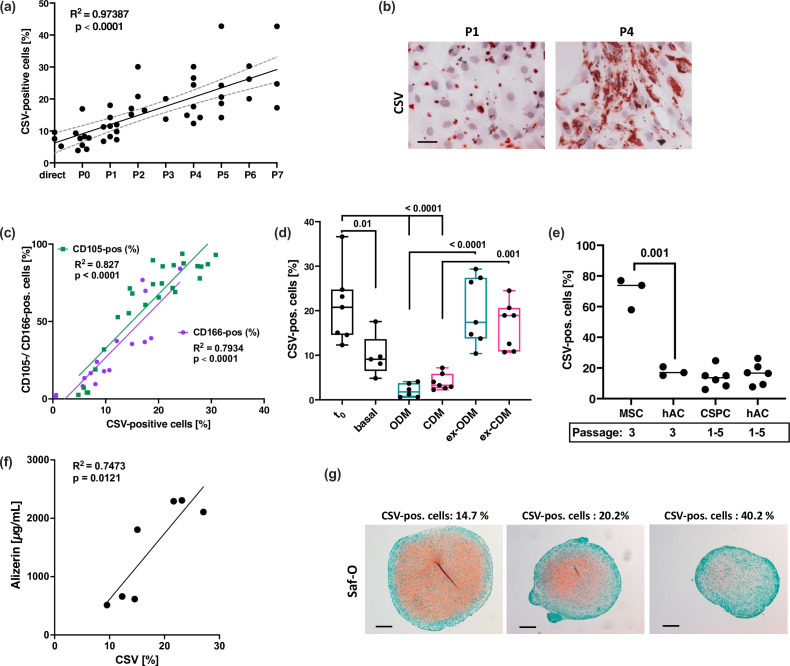


The originally published Figure 6g is shown for reference, including the reference to the initial publication:

**Figure 2:**
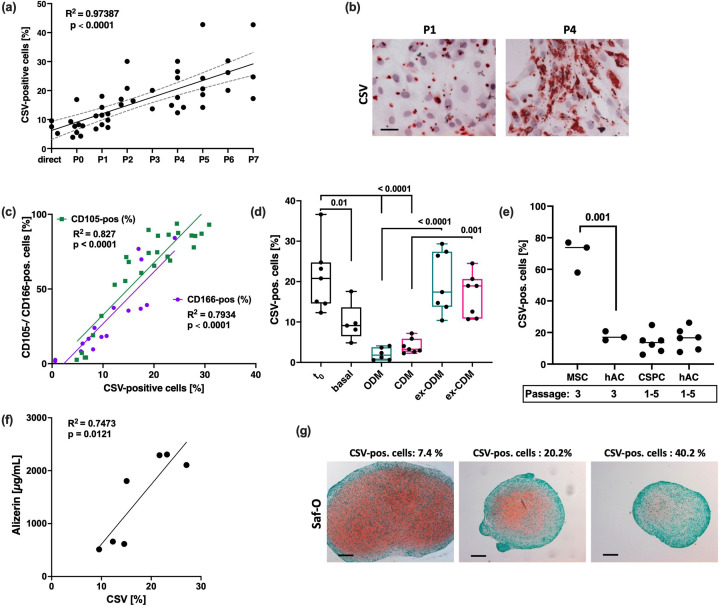


Note: The first image, depicting a Safranin-O-stained pellet after chondrogenic differentiation, has previously been published in Riegger et al., 2022. *Front Bioeng Biotechnol*; 10:965302. doi: 10.3389/fbioe.2022.965302. Licensed under CC BY 4.0, https://creativecommons.org/licenses/by/4.0/

The article has been corrected accordingly.

