# Supplementary material for: Increase of cell surface vimentin is associated with vimentin network disruption and subsequent stress-induced premature senescence in human chondrocytes
Source: eLife. 2023 Oct 19;12:e91453. doi: 10.7554/eLife.91453 (PMC10622146; doi:10.7554/eLife.91453)

Supporting information

S1: Morphology of hAC isolated from OARSI grade ≤ 1 or grade ≥ 3 tissue, w/ or w/o Doxo stimulation and after *VIM* knockdown. Left side: morphological changes upon OA, doxo-treatment (7d) and *VIM* knockdon (7d). Right side: time-dependent alteration of chondrocyte morphology after *VIM* knockdown. siNC = negative control (non-binding siRNA); siVIM = *VIM*-binding siRNA.

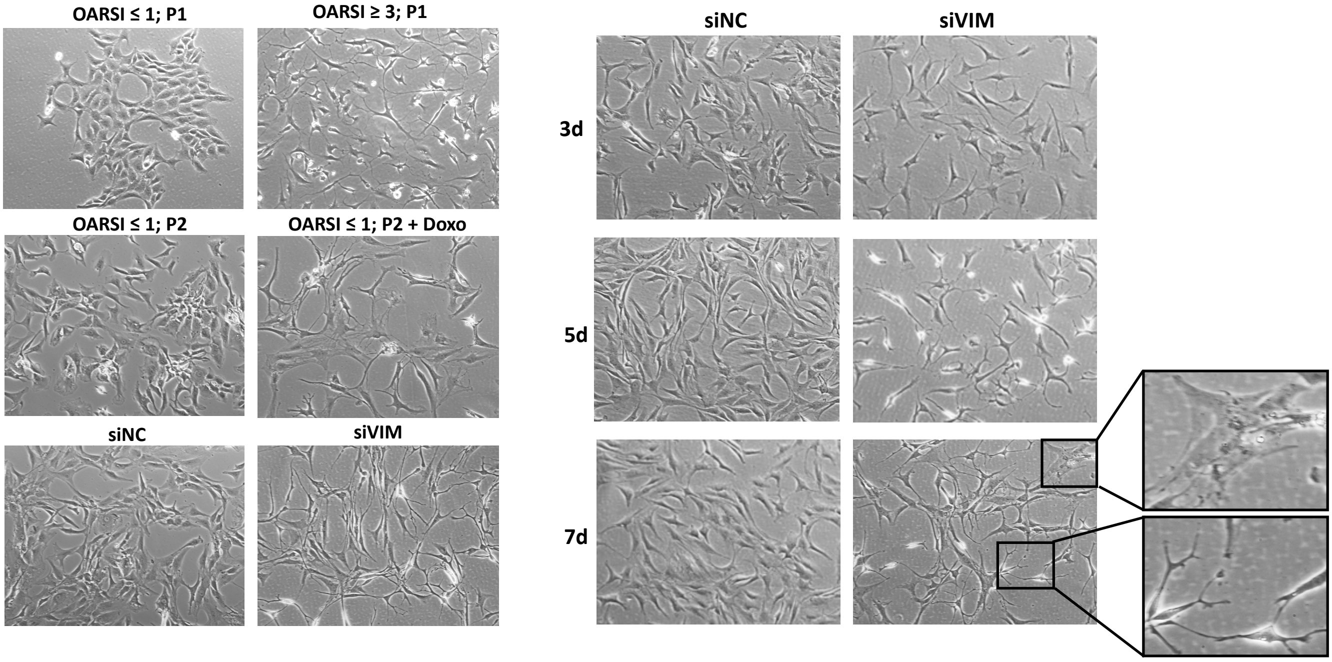

S2: Collagen II synthesis after VIM knockdown (7d).
Immunofluorescence staining of collagen II (COL2) in red. siNC = negative control (non-binding siRNA); siVIM = *VIM*-binding siRNA.

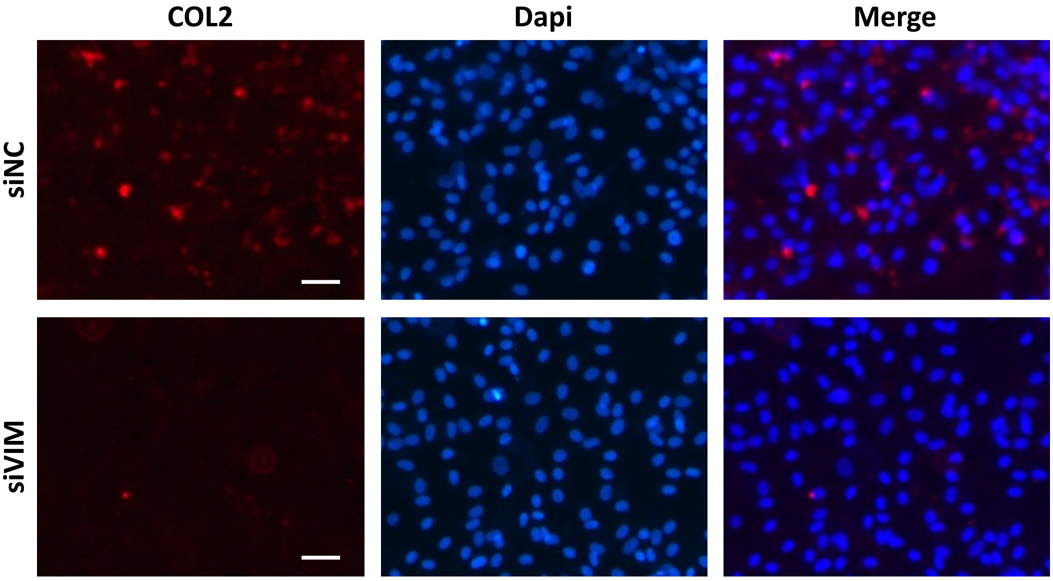

S3: Confirmation of in vitro osteogenic and chondrogenic differentiation. hAC were cultured in basal, osteogenic (ODM), or chondrogenic (CDM) medium for 28 d. After osteogenic and chondrogenic differentiation, cells were further cultured for 14 d in basal medium at sub-confluence (= ex-ODM and ex-CDM). Differentiation was confirmed by osteogenic markers (a) RUNX2 and (b) ALPL, as well as (c) alizarin red staining, or chondrogenic markers (d) ACAN and (e) COL2A1, as well as (f) Alcian blue staining. Gene expression was analysed after 28 d (= basal, ODM, CDM) and 42 d (= ex-ODM, ex-CDM).

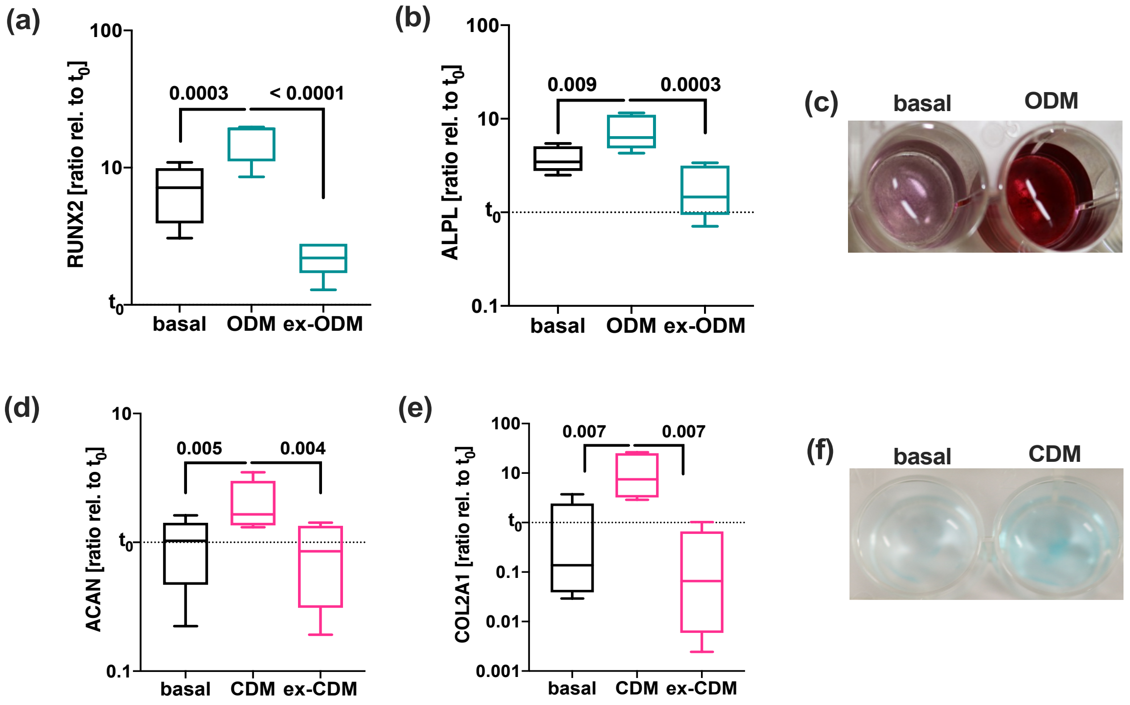

S4: Gene expression pattern of CD166, CD105, and vimentin during differentiation experiment. hAC were cultured in basal, osteogenic (ODM), or chondrogenic (CDM) medium for 28 d. After osteogenic/ chondrogenic differentiation, cells were further cultured for 14 d in basal medium at sub-confluence (= ex-ODM and ex-CDM). Gene expression of (a) CD166 (ALCAM), (b) CD105 (ENG), and (c) vimentin (VIM) was analysed after 28 d (= basal, ODM, CDM) and 42 d (= ex-ODM, ex-CDM).

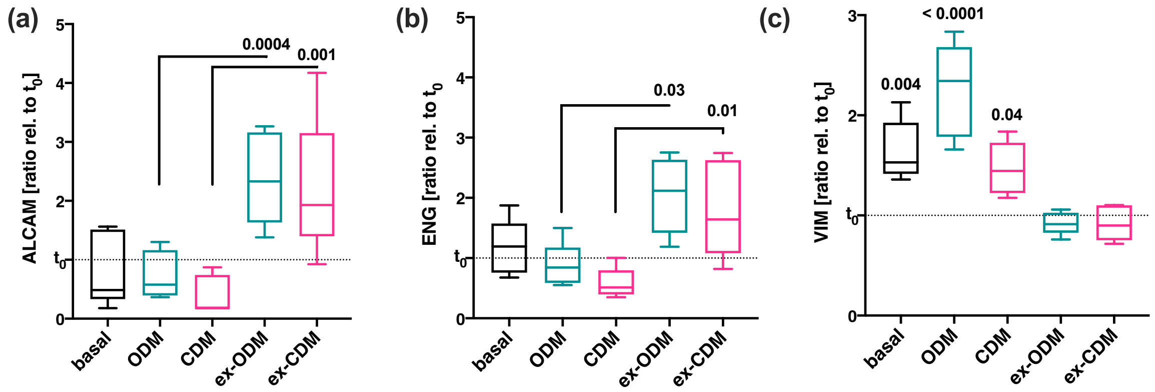

S5: Cell adhesion assay using Hela and Saos-II cells. Cancer cell lines HeLa (low CSV levels) and Saos-II (mediate/ high CSV levels) were used to confirm the influence of CSV on cell adhesion (n = 4 each cell line).

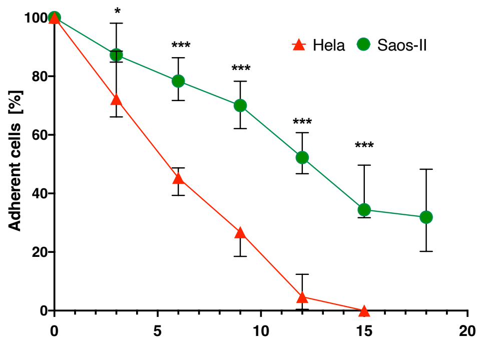

S6: Flow cytometric analysis of CSV on apoptotic hAC. To analyse presentation of CSV on apoptotic hAC, apoptosis was induced by TNFa (100 ng/ mL) and cycloheximide (CHX, 10 µg/ mL).

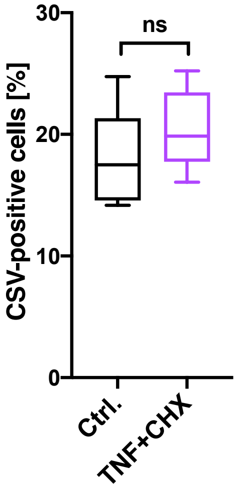

S7: Validation of the anti-CSV antibody on cancer cells. Cancer cell lines HeLa (low CSV levels) and Saos-II (mediate/ high CSV levels) were used to validate the anti-CSV antibody and the staining protocol via (a) flow cytometry and (b) immunocytochemistry.

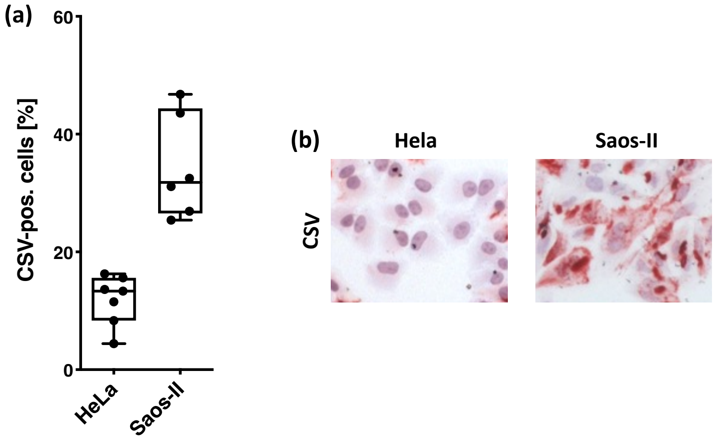

S8: Stability of CSV towards enzymes used for hAC detachment and isolation, respectively. Human articular cartilage was treated with 0.0025 % v/v collagenase (Coll) overnight (16 h) for chondrocyte isolation and analyzed by flow cytometry either immediately (Coll directly) or after 24h cultivation and detachment of adherent cells with EDTA, accutase, trypsin/ EDTA or collagenase (Coll 24h p.d.) for 10 to 20 min. CSV-positivity was determined by means of flow cytometry.

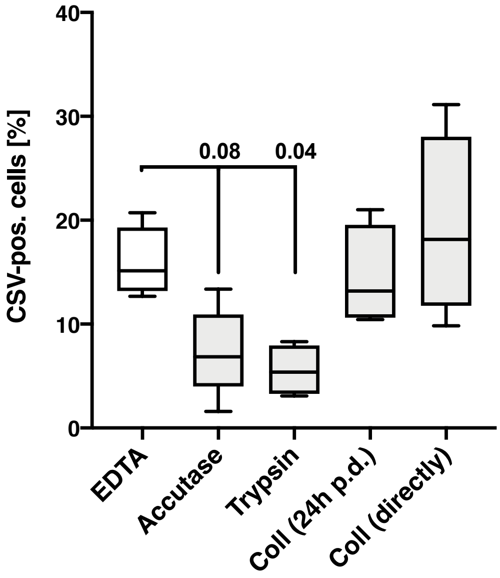

Supplement: Supplementary file 1. [file elife-91453-supp1.pdf]
